# Ultrasonographic cross-sectional area in classifying ulnar neuropathy at the elbow

**DOI:** 10.1016/j.cnp.2026.05.009

**Published:** 2026-06-05

**Authors:** Nathan J. Savage, John S. McKell

**Affiliations:** a Department of Physical Therapy, High Point University, Congdon Hall 4152, One University Parkway, High Point, NC 27268, USA; b Department of Physical Therapy, McKell Therapy Group, LLC, Orem UT84097, USA

**Keywords:** Cross-sectional area, Discriminative performance, Electrodiagnosis, Electromyography, Nerve conduction, Sonography, Ulnar neuropathy

## Abstract

**Objective:**

Ulnar neuropathy at the elbow (UNE) is a common entrapment neuropathy for which electrodiagnostic (EDX) testing is frequently used. Although multi-tiered electrophysiologic severity classifications exist, the extent to which ultrasound imaging (USI)-derived ulnar nerve cross-sectional area (CSA) provides incremental discrimination across severity categories and informs probabilistic clinical decision-making remains unclear.

**Methods:**

This retrospective analysis of prospectively collected cross-sectional data included 299 elbows from 262 patients referred for EDX testing with clinical suspicion of UNE. Elbows were classified using two multi-tiered electrophysiologic systems (Padua and Zeidman) and consolidated binary categorizations (Negative vs UNE). USI-derived ulnar nerve CSA was evaluated using receiver operating characteristic analyses and likelihood ratios to estimate post-test probability shifts across severity categories and binary classifications, accounting for clustering of bilateral elbows.

**Results:**

CSA demonstrated limited incremental discrimination between adjacent categories within multi-tiered electrophysiologic classifications, resulting in modest post-test probability shifts. In contrast, binary categorizations showed stronger discriminative performance. CSA values <6 mm^2^ identified a low probability, whereas values >15–16 mm^2^ identified a high probability of electrophysiologic abnormality suggesting UNE. Thresholds derived from Youden's index (≤8 mm^2^ and ≥ 9 mm^2^) produced smaller, non-conclusive probability shifts. Age and sex influenced electrophysiologic findings but did not materially alter CSA-based probabilistic interpretation.

**Conclusions:**

USI-derived ulnar nerve CSA does not reliably discriminate between adjacent electrophysiologic severity categories of UNE. A simplified binary interpretive framework using CSA thresholds may offer greater clinical utility.

**Significance:**

These findings support probabilistic, rather than severity-grading, use of USI when evaluating suspected UNE in patients referred for EDX testing.

## Highlights

• Electrophysiologic severity classifications show limited discriminative value.
• Binary categorizations improve clinical decision-making for ulnar neuropathy.
• Sonographic ulnar cross-sectional area provides clinically actionable thresholds.

## Graphical abstract

Cross-section area (CSA) does not reliably discriminate between categories of relative electrophysiologic severity of ulnar neuropathy at the elbow (UNE). CSA demonstrates excellent discriminative performance for consolidated binary categorizations of UNE. CSA thresholds of <6 mm^2^ or > 15 mm^2^ produces conclusive shifts in post-test probability for absence or presence of UNE, respectively. CSA thresholds of ≥9 mm^2^ or ≤ 8 mm^2^ produce only small shifts in post-test probability and should be applied cautiously.

**Figure:**
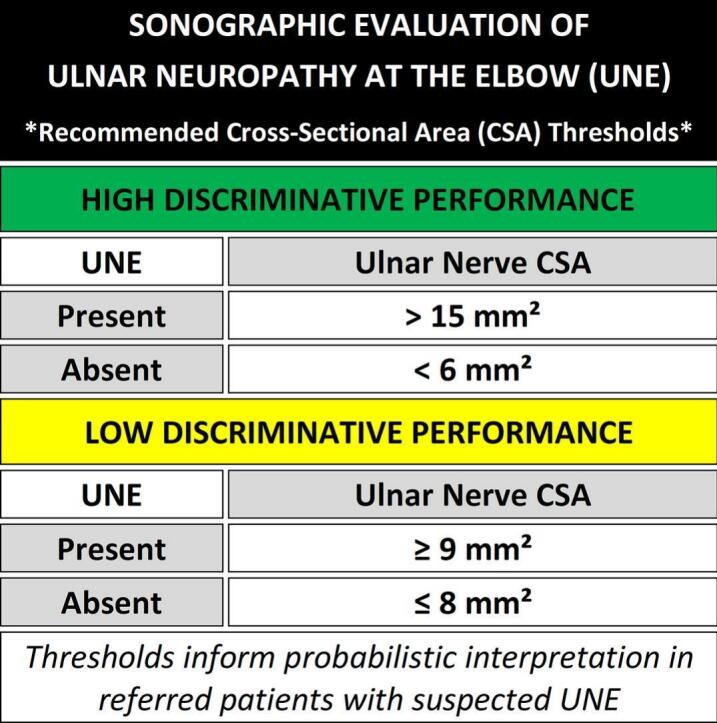

## 1 Introduction

Ulnar neuropathy at the elbow (UNE), often clinically grouped under the term cubital tunnel syndrome, is the second most common entrapment neuropathy, with prevalence estimates ranging from 2% to 6% in the general population, an incidence of approximately 25–30 per 100,000 person-years, and direct surgical treatment costs exceeding $500 million annually in the United States. (Brogan et al., 2025; Mondelli et al., 2020).

Electrodiagnostic (EDX) testing—comprising peripheral sensory and motor nerve conduction studies and needle EMG—has long been considered a reference standard for the evaluation of peripheral neuropathies; however, false-negative findings remain a concern. (Katirji, 2007; Preston and Shapiro, 2005; Dumitru, 2001; Kimura, 2001; Liveson, 2000) In patients presenting with signs and symptoms suggesting UNE, clinicians routinely employ EDX testing to evaluate for the presence of neuropathy, rule out competing neuromuscular disorders, and to inform selection of interventions based on location and severity of the nerve lesion.(Brogan et al., 2025; Savage and Heston, 2023; Yadav, 2022; Omejec and Podnar, 2020; Pelosi and Mulroy, 2019; Raeissadat et al., 2019; Pelosi et al., 2018; Halac et al., 2015; Savage et al., 2015; Savage et al., 2014; Padua et al., 2001) Importantly, electrophysiologic severity classifications describe patterns of nerve dysfunction rather than patient-reported symptom severity or functional impairment.

Multi-tiered severity classifications exist that seek to describe the relative severity of UNE based on specific electrophysiologic findings, including Padua et al. (Padua),(Padua et al., 2001) Greathouse et al.,(Greathouse et al., 2017) and Zeidman and Pandey (Zeidman).(Zeidman and Pandey, 2020) Notably, the Padua severity classification exclusively considers nerve conduction data, while the Greathouse et al. and Zeidman classifications incorporate both nerve conduction and needle EMG findings. The severity classification proposed by Greathouse et al. requires the evaluation of ulnar sensory conduction across the elbow in each of their proposed categories. Because this test is not routinely performed in our EDX laboratory—and is likely not performed routinely in many laboratories (Buschbacher et al., 2016; American Association of Electrodiagnostic Medicine, 1999)—this classification was excluded from further analysis due to limited pragmatism and generalizability in routine clinical neurophysiology practice. While EDX testing is conducted as an extension of the clinical examination in patients with symptoms suggesting neuropathy and other neuromuscular disorders, the classifications evaluated in this study are based on electrophysiologic findings only and are not intended to directly reflect clinical symptom severity or functional status.

Recently, high-resolution ultrasound imaging (USI) has emerged as an important tool for evaluating peripheral nerve morphology and mobility, as well as adjacent soft tissues and vasculature.(Pelosi et al., 2021; Carroll and Simon, 2020; Gonzalez and Hobson-Webb, 2019; Jacobson, 2018; Walker et al., 2018; Cartwright et al., 2015) Increasingly, USI is used to assist in the evaluation of patients with a clinical suspicion of neuropathy and other neuromuscular disorders and has been shown to be a convenient, safe, and cost-effective point-of-care imaging modality for a variety of neuromusculoskeletal conditions including UNE.(Becciolini et al., 2024c, Becciolini et al., 2024d, Manske et al., 2024, Shook et al., 2022, Jacobson, 2018, Pelosi et al., 2018) Most studies investigating the use of USI for the evaluation of patients with a clinical suspicion of UNE describe ulnar nerve cross-sectional area (CSA) as the most informative measurement.(Kurihara et al., 2023; Shook et al., 2022; Haj-Mirzaian et al., 2020; Aird et al., 2019; Chang et al., 2018; Jacobson, 2018) Beyond nerve enlargement, USI uniquely allows visualization of nerve mobility, focal compression sites, and surrounding soft tissue structures, which may influence interpretation of CSA measurements.

In patients with symptoms suggesting the presence of UNE, contemporary evaluation methods often include the use of USI in conjunction with EDX testing to assist in the differential diagnosis of neuropathic symptoms and to investigate competing diagnoses.(Savage and Mckell, 2024; Omejec and Podnar, 2020; Pelosi and Mulroy, 2019; Pelosi et al., 2018) This combined approach helps guide clinical decision-making by providing information regarding the presence and relative electrophysiologic severity classifications of UNE, as well as relevant nerve morphology and mobility findings, including the influence of surrounding soft tissues in the medial elbow region.

Previous studies investigating the relationship between ulnar nerve CSA and UNE severity have had mixed results;(Kurihara et al., 2023; Pelosi and Mulroy, 2019; Chang et al., 2018; Pelosi et al., 2018) however, no studies have investigated the ability of USI findings to provide incremental discrimination between consecutive categories of UNE severity across more than one EDX classification. Furthermore, no studies have evaluated the discriminative performance of ulnar nerve CSA threshold values across a continuum of prevalence values (i.e., pre-test probabilities) in patients with a clinical suspicion of UNE, which could inform clinical decision-making in the differential diagnosis and clinical management of patients with UNE.

The purpose of this investigation was to evaluate the discriminative performance of ulnar nerve CSA threshold values, using electrophysiologic findings as reference comparators across consecutive categories within electrophysiologic severity classifications and consolidated binary categorizations of UNE (i.e., “Negative” or “UNE”) in a referral-based cohort with clinical suspicion of UNE. We hypothesized that ulnar nerve CSA measurements would demonstrate limited incremental discrimination between consecutive categories of relative UNE severity and that consolidated binary categorizations would provide the greatest overall discriminative utility in patients with clinical suspicion of UNE.

## 2 Methods

### 2.1 Study design and participants

In this retrospective analysis of cross-sectional patient data, the primary outcome was the electrophysiologic categorization of UNE severity used as a reference comparator for evaluating discriminative performance rather than diagnostic accuracy. All study-related procedures were approved by the Institutional Review Board at Winston-Salem State University (IRB-FY2023–37). Because this was a retrospective review of EDX testing data, clinical examination findings and symptom severity measures were not consistently available or recorded for extraction or analysis; referral for EDX testing and USI with clinical suspicion of UNE was therefore used as the operational definition of pre-test probability.

Study participants were patients undergoing EDX and USI examinations for clinical suspicion of UNE at Therapy West Physical Therapy & Sports Medicine Centers located in Richfield and Gunnison, Utah. No asymptomatic control group was included, as all participants were referred for evaluation of symptoms and signs suggesting UNE. Participating patients provided written informed consent prior to testing, and every effort was made to ensure their rights were protected, including secure handling and de-identification of personal and health-related information.

### 2.2 Examiners

All EDX examinations were completed by two examiners (NJS and JSM), with the final EDX impression determined by the principal investigator (NJS), a Board-Certified Clinical Electrophysiologic Specialist (American Board of Physical Therapist Specialties) with over 20 years of clinical, teaching, and research experience in EDX testing. EDX examinations and final impressions were performed as part of routine clinical evaluation and were not blinded to referral information or prior clinical findings. All USI examinations were performed by a single examiner (NJS), who is Registered in Musculoskeletal® sonography (Alliance for Physician Certification & Advancement) with over 9 years of clinical, teaching, and research experience in neuromusculoskeletal USI. USI examinations and final impressions were performed as part of routine clinical evaluation.

EDX examinations were completed prior to USI examinations as part of routine clinical workflow. USI examinations and CSA measurements were performed with awareness of referral information and, in most cases, the electrophysiologic findings (i.e., the results of the EDX examinations completed by JSM were not always known at the time of USI examination).

### 2.3 Electrodiagnostic and sonographic examinations

Sierra Wave and Sierra Summit devices (Cadwell; Kennewick, WA) were used to collect all electrophysiologic data. Upper extremity nerve conduction studies were performed with patients seated and skin temperature maintained ≥32 °C. Sensory and motor nerve conduction studies followed the standardized setup and performance described by Buschbacher,(Buschbacher et al., 2016) including analysis of distal latencies, conduction velocities, and amplitudes based on normative values adjusted for patient sex, age, and height.

Needle EMG was performed with patients in the supine position using monopolar needle electrodes to evaluate insertional activity, resting activity, and volitional muscle activation for analysis of motor unit morphology and recruitment patterns. Muscles representing the C5 through T1 nerve roots and all primary peripheral motor nerves were assessed. The flexor carpi ulnaris and first dorsal interosseous muscles were routinely evaluated, while the abductor digiti minimi was included selectively to clarify the final EDX impression.

Antidromic superficial radial and median distal sensory latencies and amplitudes were obtained by stimulating the wrist and recording from the thumb over a 10-cm distance. Antidromic median and ulnar distal sensory latencies and amplitudes were obtained by stimulating the wrist and palm and recording from the middle and little fingers over 14-cm and 7-cm distances, respectively. Orthodromic median and ulnar distal motor latencies and amplitudes were obtained by stimulating the wrist and recording from the abductor pollicis brevis and abductor digiti minimi muscles, respectively, over an 8-cm distance. Orthodromic ulnar motor nerve conduction velocity across the elbow was obtained by stimulating below and above the elbow over a 10-cm segment.

Immediately following testing, the final EDX impression was determined for all elbows as part of routine clinical interpretation, designated as either “Negative” or “UNE”. In general, the presence of UNE was defined electrophysiologically by ulnar motor NCV slowing across the elbow, with or without distal ulnar sensory abnormalities (i.e., low amplitude or absent response), and with or without needle EMG abnormalities in ulnar-innervated muscles.

A 13–5 MHz linear array transducer was used for all measurements (LOGIQ e BT12; GE HealthCare, Chicago, IL, USA). Patients were positioned supine with the elbow extended off the table to allow for transducer manipulation around the medial elbow region, including adjacent arm and forearm areas. The ulnar nerve was evaluated at the medial epicondyle, immediately proximal to the medial epicondyle (triceps region), and immediately distal to the medial epicondyle (between the heads of the flexor carpi ulnaris). Entrapment site was not stratified for analysis, as CSA measurements were intentionally evaluated based on the maximal observed value to reflect routine clinical interpretation rather than site-specific diagnostic categorization. Short-axis images were obtained of the maximal ulnar nerve CSA at or near the medial epicondyle using an electronic caliper to trace the inner hyperechoic border of the epineurium. A final USI impression was determined for all elbows for descriptive comparison using the following ulnar nerve CSA threshold values: “Negative” (≤9 mm^2^) or “UNE” (>9 mm^2^).(Jacobson, 2018) These CSA thresholds were applied for descriptive comparison only and were not used to define study outcomes or reference classifications.

### 2.4 Electrodiagnostic classifications

**Fig. 1:**
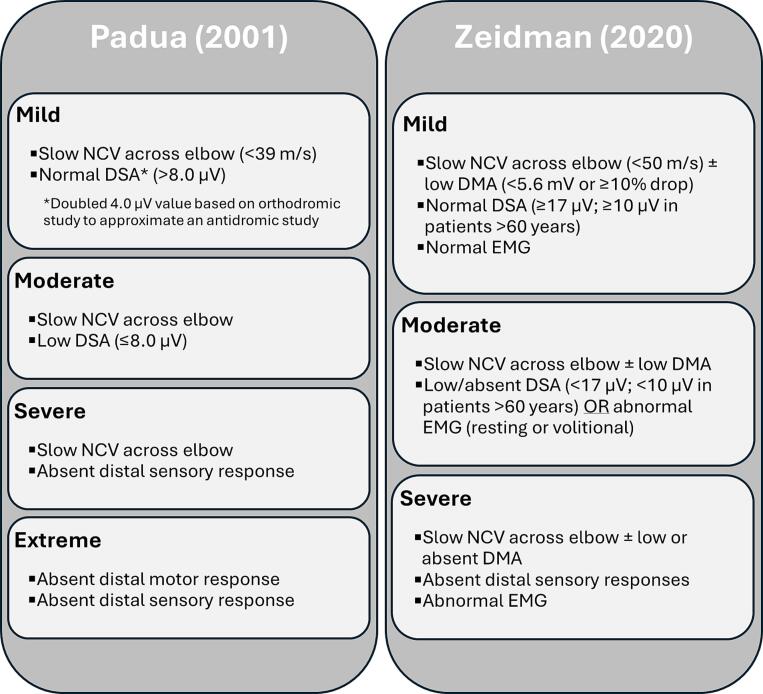
Electrophysiologic severity classification criteria for ulnar neuropathy at the elbow as reference comparators in the present analysis. NCV, nerve conduction velocity; m/s, meters per second; DSA, distal sensory amplitude; μV, microvolts; DMA, distal motor amplitude.

In addition to the final EDX impression, and for the purposes of this investigation, elbows were categorized according to UNE severity based on the electrophysiologic criteria defined in the Padua and Zeidman classifications (Fig. 1). Each classification uses different threshold values for defining abnormalities in ulnar sensory and motor responses, which also differ from the normative values described by Buschbacher(Buschbacher et al., 2016) used to determine the final EDX impression.

Elbows that did not meet strict criteria for a single severity category were classified according to the closest applicable category based on expert clinical judgement applied with predefined electrophysiologic criteria to reflect routine clinical interpretation rather than strict algorithmic classification. For statistical analysis, the four severity levels in the Padua classification and the three levels in the Zeidman classification were also analytically consolidated into binary categorizations (“Negative” or “UNE”) to match the final EDX impression.

### 2.5 Data analysis

IBM© SPSS© Statistics, version 30.0.0.0. (Armonk, NY, USA) was used for all data analyses. Descriptive statistics were used to summarize patient demographics and elbow characteristics. To account for non-independence of observations when both elbows from a single patient were included, linear mixed-effects models were used with patient specified as a random effect. This approach accounts for within-subject correlation between elbows while allowing estimation of fixed effects for EDX severity category and consolidated binary categorization. All mixed-model analyses are therefore appropriately adjusted for clustering at the patient level.

Receiver operating characteristic (ROC) curve analysis was performed for each electrophysiologic severity classification and consolidated binary categorization to estimate CSA threshold values and associated likelihood ratios (LRs) for discriminative performance within the same derivation dataset. Youden's index was calculated, and optimal LRs were identified to determine CSA thresholds that produced conclusive shifts in post-test probability.(Hosmer et al., 2013; Akobeng, 2007; McGee, 2002) Because post-test probability is influenced by disease prevalence, pre-test probabilities of 25% (unlikely), 50% (equivocal), and 75% (likely) were used to illustrate probability shifts in patients with a clinical suspicion of UNE. Independent *t*-tests were used to evaluate differences in ulnar nerve CSA based on the presence or absence of observed ulnar nerve subluxation/dislocation and self-reported diabetes comorbidity. These CSA thresholds were applied for descriptive comparison only and were not used to define study outcomes or reference classifications.

## 3 Results

Data were collected from December 2019 through July 2023 on 262 patients (mean age 52.6 ± 18.0 years; mean height 67.8 ± 3.9 in.; 55% male). These patients were referred for EDX and USI examinations with a clinical suspicion of UNE (69% Medical Doctor or Osteopath; 29% Physician Assistant or Nurse Practitioner; 2% direct access). A total of 299 elbows (53% left-sided) (Ashworth et al., 2020) were included for analysis.

EDX examinations were completed by two examiners (76% NJS; 24% JSM), and the final EDX impression for all elbows was determined by the principal investigator (NJS). Based on ulnar sensory and motor nerve conduction findings using Buschbacher's normative values (Buschbacher et al., 2016) and needle EMG results, the final EDX impression categorized 140 elbows as “Negative” (47%) and 159 elbows as “UNE” (53%).

After consideration of ulnar nerve morphology and mobility at or near the medial epicondyle, the final USI impression for descriptive comparison categorized 150 elbows as “Negative” (50.2%) and 149 elbows as “UNE” (49.8%). The prevalence of ulnar nerve subluxation/dislocation was 24% (72 of 299 elbows), and the prevalence of diabetes was 15% (45 of 299 elbows).

Shapiro–Wilk testing indicated that ulnar nerve CSA deviated from normality; however, given the large sample size and robustness of ROC curve analysis to violations of normality, parametric analyses were retained.

### 3.1 Severity classification of ulnar neuropathy at the elbow

**Fig. 2:**
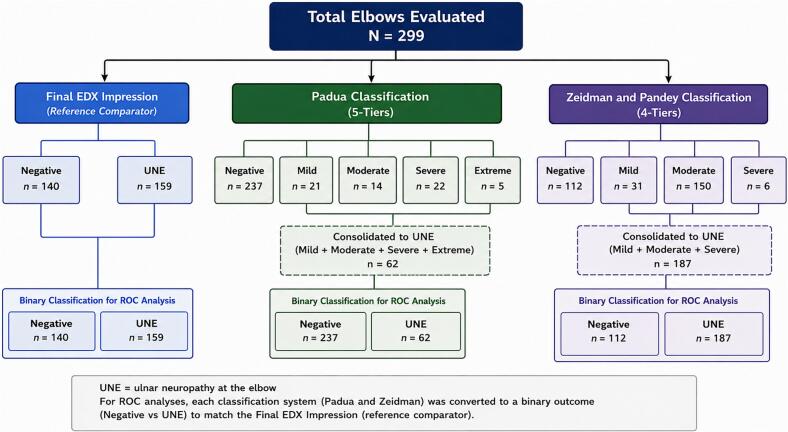
Analytic workflow of categorizing the 299 elbows across electrophysiologic classifications and how multi-tiered classifications were consolidated into binary reference categorizations for ROC curve analyses. ROC, receiver operating characteristic.

The analytic classification workflow used in this study is summarized in Fig. 2. All 299 elbows were categorized in parallel using three electrophysiologic reference systems: the final EDX impression, the Padua classification, and the Zeidman and Pandey classification. For analytic purposes, multi-tiered Padua and Zeidman classifications were consolidated into binary reference categorizations (Negative vs UNE) to align with the final EDX impression prior to CSA-based ROC and LR analyses. Chi-square analysis comparing consolidated binary electrophysiologic categorizations with the final USI impression demonstrated significant association across all classifications (Final EDX Impression: χ^2^ = 76.6, *P* < .001; Padua: χ^2^ = 47.3, P < .001; Zeidman: χ^2^ = 163.1, P < .001).

For descriptive comparison, categorical concordance between USI and electrophysiologic classifications ranged from 60% to 80% for the “Negative” category and from 68% to 89% for the “UNE” category across classifications. These comparisons were intended to describe concordance patterns between classification frameworks rather than to assess diagnostic accuracy.

### 3.2 Ulnar nerve cross-sectional area

Independent *t*-tests revealed no significant difference in ulnar nerve CSA between elbows with and without observed ulnar nerve subluxation/dislocation (11.0 ± 6.3 mm^2^ vs 10.1 ± 4.9 mm^2^, respectively; *P* = .198). Similarly, no significant difference in CSA was observed between elbows from patients with and without self-reported diabetes (11.2 ± 3.9 mm^2^ vs 10.2 ± 5.4 mm^2^, respectively; *P* = .255).

Sonographic findings for each electrophysiologic severity classification are summarized in Table 1. ROC curve analysis and associated LRs describing discriminative performance for ulnar nerve CSA threshold values across all electrophysiologic severity classifications and consolidated binary categorizations of UNE are summarized in Table 2.

**Table 1:** Ulnar nerve CSA by electrophysiologic severity classification of ulnar neuropathy at the elbow.

Classification	Severity	N	CSA (mm^2^) Mean ± SD
Padua et al.	Negative	237	9.09 ± 4.00
	Mild	21	14.33 ± 7.77
	Moderate	14	13.64 ± 3.46
	Severe	22	16.50 ± 5.85
	Extreme	5	16.20 ± 10.76
Zeidman and Pandey	Negative	112	7.39 ± 3.48
	Mild	31	9.90 ± 3.54
	Moderate	150	12.44 ± 5.30
	Severe	6	14.83 ± 10.19

CSA, cross-sectional area; mm^2^, millimeters squared; SD, standard deviation.

**Table 2:** Discriminative performance of ulnar nerve CSA threshold values based on receiver operating characteristics curve analysis and likelihood ratios for UNE.

Classification	Category comparison	AUC (95% CI)	CSA (mm^2^)	+LR / -LR	Optimal CSA (mm^2^)	Optimal +LR / -LR
(A) Multi-Tiered Classifications						
Padua et al.	Mild v Neg	^†^0.760 (0.662, 0.858)	9.5	2.16 / 0.24	19.5 / 5.5	9.52 / 0.00
	Mod v Mild	0.573 (0.377, 0.769)	12.5	1.83 / 0.37	NA	NA
	Severe v Mod	0.649 (0.468, 0.831)	16.5	2.86 / 0.69	NA	NA
	Ext v Severe	0.477 (0.123, 0.831)	18.5	2.20 / 0.73	NA	NA
Zeidman and	Mild v Neg	^†^0.739 (0.650, 0.827)	8.5	2.58 / 0.47	NA / 5.5	NA / 0.08
Pandey	Mod v Mild	^†^0.660 (0.563, 0.758)	12.5	3.26 / 0.67	NA	NA
	Severe v Mod	0.519 (0.219, 0.820)	18.5	3.33 / 0.74	25.0 / NA	8.35 / NA

(B) Consolidated binary categorizations						
Final EDX Impression	UNE v Neg	†0.810 (0.760, 0.859)	8.5	2.77 / 0.29	16.5 / 5.5	9.00 / 0.12
Binary Padua	UNE v Neg	†0.809 (0.748, 0.870)	12.5	4.10 / 0.39	20.5 / 5.5	8.69 / 0.15
Binary Zeidman	UNE v Neg	†0.807 (0.754, 0.859)	8.5	2.95 / 0.35	15.5 / 5.5	7.74 / 0.11

CSA, cross-sectional area; UNE, ulnar neuropathy at the elbow; Neg, negative; Mod, moderate; Ext, extreme; AUC, area under the curve; CI, confidence interval; mm^2^, millimeters squared; +LR, positive likelihood ratio; −LR, negative likelihood ratio; EDX, electrodiagnostic.

† *P* < .05.

Across the Padua and Zeidman classifications, ulnar nerve CSA demonstrated area under the curve (AUC) values ranging from no discrimination to acceptable discriminative performance (0.477–0.760).(Hosmer et al., 2013) Youden's index identified CSA thresholds associated with small shifts in post-test probability,(McGee, 2002) with positive (+) LRs ranging from 1.83 to 3.33 and negative (−) LRs ranging from 0.74 to 0.24. Optimal LRs producing larger shifts in post-test probability were observed only when comparing “Mild” versus “Negative” categories in both classifications.

**Fig. 3:**
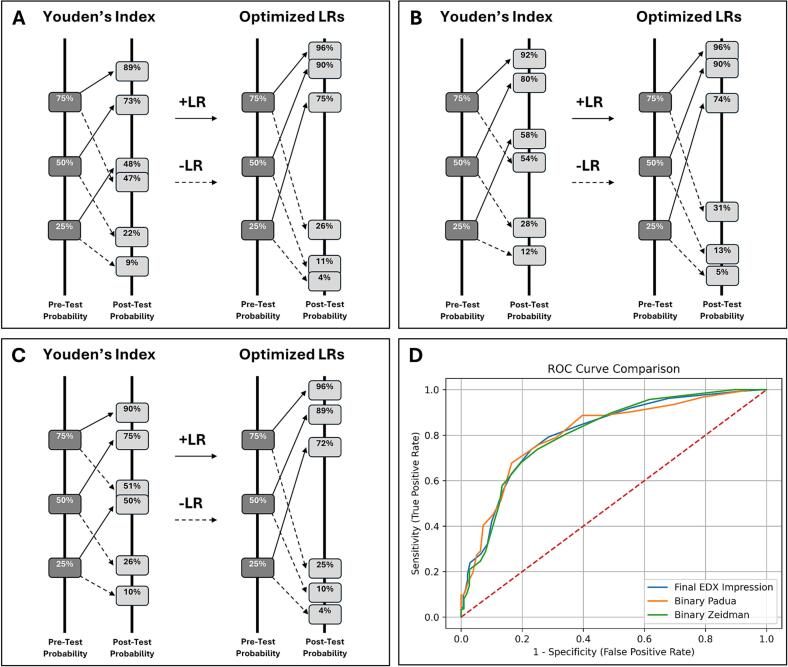
Shifts in post-test probability of ulnar neuropathy at the elbow based on ulnar nerve CSA threshold values across consolidated binary electrophysiologic reference categorizations: (A) Final EDX Impression, (B) Binary Padua, (C) Binary Zeidman. Panel (D) displays receiver operating characteristic (ROC) curves derived from the same referral-based cohort and used to estimate LRs and associated probability shifts rather than to establish diagnostic accuracy. CSA, cross-sectional area; EDX, electrodiagnostic; LRs, likelihood ratios; +LR, positive likelihood ratio; −LR, negative likelihood ratio.

In contrast, CSA threshold values across consolidated binary categorizations demonstrated excellent discriminative performance (AUC = 0.807–0.810).(Hosmer et al., 2013) Optimal LRs produced clinically meaningful shifts in post-test probability, with +LRs ranging from 7.74 to 9.00 and -LRs ranging from 0.15 to 0.11 (Fig. 3).

Overall, CSA thresholds based on optimal -LRs identifying a low probability of electrophysiologic abnormality suggesting UNE were consistent across consolidated binary categorizations at <6 mm^2^. Thresholds identifying a high probability of abnormality were most consistent in categorizations incorporating needle EMG findings, with values >15–16 mm^2^.

Youden's index identified CSA thresholds of ≤8 mm^2^ and ≥ 9 mm^2^ across all consolidated binary categorizations; however, the associated LRs produced only small shifts in post-test probability and did not reach conventional thresholds for conclusive diagnostic interpretation. In contrast, CSA thresholds derived from optimal -LRs were more consistent across categorizations, with values <6 mm^2^ identifying a low probability of electrophysiologic abnormality suggesting UNE. Thresholds associated with optimal +LRs were more variable ranging from >15 mm^2^ to >20 mm^2^ and demonstrated the greatest internal agreement in categorizations incorporating needle EMG findings, with thresholds of >16 mm^2^ and > 15 mm^2^, respectively (Table 3).

**Table 3:** Distribution of elbows and discriminative performance using recommended binary categorizations of UNE.

CSA	Count (%)	Presence of UNE	Shift in post-test probability
(A) Optimal likelihood thresholds			
<6 mm^2^	51 (17%)	Low probability	Conclusive
>15 mm^2^	42 (14%)	High probability	Conclusive
Unclassified	206 (69%)	Equivocal	NA

(B) Youden's index thresholds			
≤8 mm^2^	133 (44%)	Low probability	Small
≥9 mm^2^	166 (56%)	High probability	Small

UNE; ulnar neuropathy at the elbow; CSA, cross-sectional area; mm^2^, millimeters squared; NA, not applicable.

### 3.3 Additional analyses

Sex-specific analysis demonstrated significant differences in CSA between men and women (*t* = 3.9, df = 297, *P* < .001), with men exhibiting larger CSA values on average. Chi-square analyses revealed significant sex differences in prevalence of UNE across all consolidated binary categorizations (Final EDX Impression: χ^2^ = 41.7, *P* < .001; Padua: χ^2^ = 6.66, *P* = .010; Bland: χ^2^ = 54.4, P < .001), with men demonstrating a higher proportion of “UNE” categorizations. Men also had a higher prevalence of ulnar nerve subluxation/dislocation (χ^2^ = 4.9, *P* = .026) and diabetes (χ^2^ = 8.4, *P* = .004).

Age category analysis similarly demonstrated significant differences in CSA between patients <50 years and those ≥50 years (*t* = −4.48, df = 297, *P* < .001), with larger CSA values observed in the older group. Patients ≥50 years also demonstrated a higher prevalence of UNE across all consolidated binary categorizations (Final EDX Impression: χ^2^ = 55.0, P < .001; Padua: χ^2^ = 17.9, *P* = .010; Bland: χ^2^ = 44.8, P < .001), while younger patients more frequently exhibited ulnar nerve subluxation/dislocation (χ^2^ = 6.7, P = .010) and older patients more frequently reported diabetes (χ^2^ = 23.4, P < .001).

These findings indicate that sex and age influence ulnar nerve size and electrophysiologic categorization and should be considered when interpreting CSA threshold values in referred patients with a clinical suspicion of UNE.

## 4 Discussion

### 4.1 Clinical implications

This study evaluated the discriminative performance of ulnar nerve CSA, as measured by USI, using electrophysiologic classifications as reference comparators rather than as indicators of clinical severity. This investigation is unique in that it evaluated ulnar nerve CSA across existing electrophysiologic severity classifications of UNE, including those most commonly used in clinical neurophysiologic practice.(Zeidman and Pandey, 2020; Padua et al., 2001) Extensive ROC curve analysis demonstrated limited discriminative performance of ulnar nerve CSA measurements, particularly when applied to multi-tiered electrophysiologic severity classifications. High variability across multi-tiered severity classifications resulted in only modest shifts in post-test probability, indicating limited incremental value for clinical decision-making. (Hosmer et al., 2013; Akobeng, 2007; McGee, 2002).

In contrast, ROC curve analysis demonstrated strong discriminative performance of ulnar nerve CSA for identifying a low probability of electrophysiologic abnormality suggesting UNE when severity classifications were consolidated into binary reference categories within this referral-based cohort. These threshold values produced conclusive shifts in post-test probability within this referral-based cohort, informing clinical decision-making and the differential diagnostic process.(Hosmer et al., 2013, Akobeng, 2007, McGee, 2002) Ulnar nerve CSA threshold values for identifying a high probability of electrophysiologic abnormality suggesting UNE were more variable, but showed close agreement among electrophysiologic categorizations that included needle EMG findings. These findings were consistent across subgroup analyses, as additional analyses revealed no significant differences in CSA based on the presence of ulnar nerve subluxation/dislocation or self-reported diabetes comorbidity in this sample.

The results of this investigation enhance the evaluation of patients with a clinical suspicion of UNE by providing preliminary but clinically relevant insights supporting the use of USI as a complementary evaluative tool in clinical neurophysiology. Unlike much of the existing literature on USI in suspected UNE, this study explicitly evaluated discriminative performance using LRs and associated shifts in post-test probability. This approach aligns with evidence-based diagnostic reasoning and supports the use of USI as a complementary evaluative tool in clinical neurophysiology practice. (Hosmer et al., 2013; McGee, 2002; Akobeng, 2007).

In this context, when the clinical examination and/or electrophysiologic findings are equivocal or suggest a lower likelihood of UNE, ulnar nerve CSA threshold values capable of producing conclusive shifts in post-test probability are particularly valuable in patients with a clinical suspicion of UNE referred for EDX testing. Conversely, when the clinical examination and/or electrophysiologic findings are strongly suggestive of UNE, CSA threshold values producing only small shifts in post-test probability *may* be used cautiously but should not be relied upon as a confirmatory test. Importantly, these findings do not support the use of ulnar nerve CSA to stage or grade relative electrophysiologic severity, but rather to inform probabilistic reasoning when diagnostic uncertainty remains.

### 4.2 Comparison with prior literature

Comparison of these findings with those of Chang et al.(Chang et al., 2018) reveals several similarities. While Chang et al. recommended a CSA threshold value of 10 mm^2^, they also acknowledged that diagnostic criteria for UNE were not uniform across the studies they included, CSA measurement protocols varied, and the mean age in the patient group was higher than in the control group. These factors—particularly given the age-related differences in CSA observed in the present cohort—may explain why their proposed threshold values were lower than those identified in this study for generating conclusive shifts in post-test probability.

Other investigations evaluating ulnar nerve CSA in suspected UNE have similarly reported substantial variability in proposed threshold values, reflecting differences in reference standards, patient selection, measurement location, and analytic approach. Few prior studies have explicitly examined LRs or post-test probability shifts, limiting direct comparison with probabilistic interpretations derived from the present findings.(Pelosi and Mulroy, 2019; Pelosi et al., 2018)

### 4.3 Limitations

Several limitations should be acknowledged. First, all USI examinations were conducted by a single examiner who was not blinded to the majority of electrophysiologic findings, which may introduce bias in CSA measurement. However, having a single examiner also ensured methodological consistency and likely strengthened internal validity. Second, systemic medical conditions (e.g., diabetes) were identified via patient self-report without laboratory confirmation, which may have introduced variability in peripheral nerve health classification. Clinical symptom severity and functional status were not systematically captured, limiting the ability to evaluate relationships between CSA, electrophysiologic findings, and patient-reported outcomes. All participants were referred with clinical suspicion of UNE, resulting in an enriched sample with elevated pre-test probability; accordingly, LR and post-test probability shifts observed in this study should be interpreted within the context of referral-based evaluation rather and purely population-based screening.

Additionally, CSA measurements were analyzed based on maximal nerve enlargement irrespective of specific entrapment location, which may obscure site-specific structure–function relationships but reflects routine clinical interpretation in EDX laboratories. CSA threshold values may differ according to the specific site of ulnar nerve entrapment (e.g., retroepicondylar groove versus humeroulnar aponeurotic arcade), which represents an important advantage of USI but was evaluated in the present analysis due to site-agnostic CSA aggregation.

## 5 Conclusions

The use of ulnar nerve CSA threshold values to categorize the relative severity of UNE is not supported by the present data, as this approach does not produce consistent or clinically meaningful shifts in post-test probability. Instead, the present findings support consideration of a simplified binary interpretive approach using CSA threshold values of <6 mm^2^ to identify a low probability of electrophysiologic abnormality suggesting UNE and > 15 mm^2^ to identify a high probability of electrophysiologic abnormality suggesting UNE (Table 3). These thresholds produced conclusive shifts in post-test probability and may aid clinicians in the evaluative and differential diagnostic process across a range of pre-test probabilities in patients with clinical suspicion of UNE.

Additionally, CSA threshold values of ≤8 mm^2^ and ≥ 9 mm^2^ may be considered in select clinical circumstances when UNE is deemed more likely based on clinical and/or electrophysiologic findings; however, these values produce only modest shifts in post-test probability and should be applied cautiously and not interpreted as confirmatory. These findings were consistent across the electrophysiologic classifications examined.

Future research should refine the role of USI in the evaluation, differential diagnosis, and clinical management of patients with UNE, including external validation in independent and asymptomatic control populations and incorporation of clinical severity measures.

## Declaration of generative AI and AI-assisted technologies in the manuscript preparation process

During the preparation of this work the authors used Microsoft Copilot to support language refinement, formatting, and organization of the manuscript text. After using this tool, the authors reviewed and edited the content as needed and take full responsibility for the content of the published article.

## Author contributions

Nathan J. Savage conceived and designed the study, performed all ultrasound examinations, contributed to electrodiagnostic examinations, conducted data analysis, interpreted the results, and drafted the manuscript.

John S. McKell contributed to study design, performed electrodiagnostic examinations, assisted with data interpretation, and critically reviewed the manuscript for important intellectual content.

Both authors approved the final version of the manuscript and agree to be accountable for all aspects of the work.

## Declaration of competing interest

The authors declare that they have no known competing financial interests or personal relationships that could have appeared to influence the work reported in this paper. This research received no external funding.
